# Magnitude of tuberculosis and its associated factors among under-five children admitted with severe acute malnutrition to public hospitals in the city of Dire Dawa, Eastern Ethiopia, 2021: multi-center cross-sectional study

**DOI:** 10.1016/j.ijregi.2022.04.008

**Published:** 2022-04-28

**Authors:** Kendalem Asmare Atalell, Ribka Nigatu Haile, Masresha Asmare Techane

**Affiliations:** a Department of Pediatrics and Child Health Nursing, School of Nursing, College of Medicine and Health Sciences, University of Gondar, Gondar, Ethiopia; b School of Nursing, College of Health Sciences, Woldia University, Woldia, Ethiopia

**Keywords:** Associated factors, Ethiopia, Prevalence, Severe acute malnutrition, Tuberculosis, Under-five children, AIDS, acquired immune deficiency syndrome, BCG, Bacillus Calmette–Guérin, BMI, body mass index, HIV, human immunodeficiency virus, MUAC, mid-upper arm circumference, SAM, severe acute malnutrition, SDGs, Sustainable Development Goals, WFH, weight-for-height

## Abstract

**Objective:**

The aim of this study was to assess the prevalence of tuberculosis (TB) and its associated factors among children under 5 years of age with severe acute malnutrition.

**Methods:**

A multi-center, institution-based, retrospective cross-sectional study was conducted at public hospitals in Dire Dawa City Administration, Eastern Ethiopia from January 1, 2018 to December 30, 2020. A binary logistic regression model was fitted to identify factors associated with the prevalence of TB.

**Results:**

The overall prevalence of TB among children under 5 years of age admitted with severe acute malnutrition to public hospitals in the city of Dire Dawa, Eastern Ethiopia was 10.39% (95% confidence interval (CI) 7.61–13.73%). Repeated admission (adjusted odds ratio (AOR) 2.5, 95% CI 1.08–6.07), a TB contact history (AOR 3.58, 95% CI 1.21–10.6), pneumonia (AOR 2.8, 95% CI 1.29–6.23), stage IV HIV/AIDS (AOR 4.41, 95% CI 1.29–15.13), and being immunized (AOR 0.19, 95% CI 0.08–0.43) were variables significantly associated with the prevalence of TB.

**Conclusions:**

The results of this study showed that the prevalence of TB among under-five children with severe acute malnutrition was high. The prevalence of TB was associated with having HIV/AIDS, having pneumonia, having a TB contact history, admission status, and immunization status. Integrated TB prevention and screening strategies with nutritional rehabilitation care should be implemented.

## Highlights

• The prevalence of tuberculosis (TB) among children with severe acute malnutrition was 10.4%.
• Children with HIV/AIDS, pneumonia, and a contact history were at risk of developing TB.
• Immunization was found to be the predictor of TB among children with severe acute malnutrition.
• Integrated TB prevention with nutritional rehabilitation care is recommended.

## 1 Introduction

Tuberculosis (TB), caused by *Mycobacterium tuberculosis* complex, is currently the second leading cause of death from an infectious disease after COVID-19 (Munthali et al., 2017, Shakoor and Mir, 2022). According to the World Health Organization (WHO) 2021 report, 5.8 million new TB cases and 1.3 million TB deaths occurred during the year 2020 (WHO, 2021). One million children become ill with TB every year, which represents 10% of all TB cases (Grobusch and Kapata, 2018).

According to the first population-based national TB prevalence survey in Ethiopia covering the period from 2010 to 2011, the prevalence of smear-positive TB was 108/100 000 population, and that of bacteriologically confirmed TB was 277/100 000 population (Kebede et al., 2014).

TB and malnutrition have synergistic effects. TB mortality is increased in children with under-nutrition, particularly in children with severe acute malnutrition (SAM) (Munthali et al., 2017; Bjune et al., 2006; Organization, 2013). On the other hand, children with TB mostly manifest malnutrition due to the loss of appetite that occurs with the disease (Bhargava et al., 2014).

Studies have been conducted worldwide to assess the prevalence of TB among children with SAM and have reported prevalence rates of 4.67% in Nepal (Thakur et al., 2020), 10.34% (S et al., 2019) and 5.6% (Payghan et al., 2013) in India, 36.9% in Pakistan (Khalil et al., 2020), and 1.58% in Zambia (Munthali et al., 2017). In Sub-Saharan Africa, there is a higher proportion of overall pediatric TB cases, with a projection of 20% (Glaziou et al., 2016). The incidence of TB among children with SAM in Ethiopia was recently reported as 4 per 100 person-months (AYNALEM et al., 2020). Although a limited number of studies have indicated a higher prevalence of TB in children with malnutrition, there remains a lack of evidence for this. Understanding the magnitude of TB among children who are severely malnourished is important for the development of evidence-based interventions to reduce TB in children with malnutrition.

Several risk factors for TB among severely malnourished children have been identified, such as younger age, female sex, urban residence, TB contact history, comorbidities, lack of immunizations, lack of exclusive breastfeeding, and late start of complementary feeding (Khalil et al., 2020; Munthali et al., 2017; Payghan et al., 2013; Attah et al., 2018; AYNALEM et al., 2020; Chen et al., 2013; Chisti et al., 2014).

Globally, efforts have been made to control TB in order to achieve the WHO End TB Strategy and Sustainable Development Goals (SDGs), which aim to reduce TB death and incidence by 90% and 80%, respectively, by 2030 (Christof et al., 2020; Organization, 2015). In line with this, Ethiopia has also planned to end all preventable child deaths by 2035 (Shiferaw et al., 2018). Even though a slight reduction in TB prevalence worldwide has been observed, it remains a major public health problem, especially in children with SAM (Zwerling, 2020). Thus, the aim of this study was to assess the prevalence and associated factors of TB in children under 5 years of age with SAM, which is essential to inform policymakers regarding the need to have integrated TB screening and prevention strategies in all nutritional rehabilitation centers.

## 2 Methods

### 2.1 Study design and setting

This multi-center, institution-based, cross-sectional study was conducted among under-five children with SAM admitted to public hospitals in the city of Dire Dawa, Eastern Ethiopia between January 1, 2018 and December 30, 2020. Dire Dawa City Administration is among the two-city administrations in Ethiopia, which is located 520 km east of Addis Ababa (the capital of Ethiopia). There are two public hospitals in the city of Dire Dawa, i.e. Dil Chora Referral Hospital and Sabiyan General Hospital, at which the study was conducted.

### 2.2 Study participants and sampling

The source population of this study was under-five children with SAM attending public hospitals in Dire Dawa City Administration, Eastern Ethiopia between January 2018 and December 2020. The sample size was calculated using a single population proportion formula, assuming *Z*_α/2_ at a 95% confidence interval (CI) (1.96), a margin of error (*w*) of 5%, proportion (*P*) of 50%, and a 10% non-response rate; a total sample size of 422 was calculated to be required. Study participants were proportionately allocated to each hospital based on the number of children admitted with SAM, and for each of the 3 years in each hospital. Within the 3-year study period, 1000 children with SAM were admitted to Dil Chora Referral Hospital and 400 children with SAM were admitted to Sabiyan General Hospital. Thus, the proportional allocation was 120 children from Sabiyan General Hospital and 302 from Dil Chora Referral Hospital. The sample in each hospital was also proportionally allocated for each of the 3 years. The individual sample in each year within each hospital was selected using a simple random sampling technique.

### 2.3 Variable definitions

For children aged >6 months, severe acute malnutrition was defined as the presence of nutritional edema (bilateral pitting edema) or severe wasting (mid-upper arm circumference (MUAC) <11.5 cm or a weight-for-height (WFH)/weight-for-length (WFL) <−3 *z*-score). For children aged <6 months, severe acute malnutrition was defined as the presence of bilateral pitting edema (+, ++, or +++), WFL <−3 *z*-score, medical complications, recent weight loss or failure to gain weight, and ineffective feeding.

TB was the outcome variable; TB was considered present if there was a diagnosis of confirmed TB in the child's medical records signed by a physician.

### 2.4 Data collection tools and procedures

Data were extracted from the medical records of the children using a structured data extraction checklist adapted from previous studies reported in the literature. The checklist comprised sociodemographic characteristics, comorbidities, and care and treatment-related characteristics. Four BSc nurses with experience working in nutritional rehabilitation centers were recruited for the data collection. Two days of training were given to the data collectors on how to extract the data from the medical records. The medical records of the included children were retrieved and the data were carefully extracted.

### 2.5 Statistical analysis

The data were entered into EpiData version 4.2 and exported to Stata version 16 for cleaning, coding, and analysis. Descriptive statistics were computed and presented using texts, tables, charts, and graphs. Model fitness was checked using the Hosmer–Lemeshow test, which gave a *P*-value of 0.69, indicating a good fit of the model. Chi-square assumption and multicollinearity were also checked before running the logistic regression. Binary logistic regression analysis was fitted to identify factors associated with the prevalence of TB among children with SAM.

Variables with a *P*-value <0.25 in the bivariate analysis were fitted into the multivariate logistic regression. The adjusted odds ratio (AOR) was calculated and used as a measure of the association at the 95% confidence level. Variables with *P*-values <0.05 in the multivariate logistic regression analysis were identified as statistically significant.

## 3 Results

### 3.1 Sociodemographic characteristics

The medical records of a total of 422 children were reviewed, of which eight (1.9%) were excluded from the analysis due to incomplete data. The data of 414 children under 5 years of age were included in the analysis, giving a response rate of 98.1%. The median age of the study participants was 12 months; 262 (63.29%) of the participants were <24 months of age. More than half (56.28%) of the participants were male and nearly two-thirds (64.01%) resided in urban areas (Table 1).

**Table 1:** Sociodemographic and admission characteristics of children under 5 years of age admitted with severe acute malnutrition to public hospitals in the city of Dire Dawa, Eastern Ethiopia, 2021 (*N* = 414)

Variables	Categories	Frequency	Percentage
Age	<24 months	262	63.29%
	≥24 months	152	36.71%
Sex	Male	233	56.28%
	Female	181	43.72%
Residence	Urban	265	64.01%
	Rural	149	35.99%
Admission status	New	338	81.64%
	Repeat	76	18.36%
Presence of edema	No	300	72.46%
	Yes	114	27.54%
Appetite test at admission	Fail	341	82.37%
	Pass	73	17.63%
Exclusive breastfeeding	No	152	36.71%
	Yes	262	63.29%
Exclusive breastfeeding duration	No EBF	152	36.71%
	EBF for 1–3 months	34	8.21%
	EBF for 4–6 months	176	42.51%
	EBF for 7–12 months	52	12.56%
TB contact history	No	388	93.72 %
	Yes	26	6.28%
Immunization status	Not immunized	118	28.5%
	Immunized	296	71.5%
Level of consciousness	Conscious	338	81.64%
	Unconscious	76	18.36%

EBF, exclusive breastfeeding.

### 3.2 Comorbidities

Regarding comorbidities, the most common was diarrhea (*n* = 256, 61.84%), followed by pneumonia (*n* = 176, 42.51%). Vomiting, severe anemia, hyperthermia, sepsis, and superficial infection were identified in 28.74%, 26.81%, 22.95%, 13.29%, and 7.73% of the children, respectively (Table 2). Slightly more than a quarter (27.54%) of the study participants had edema during admission. Twenty-six (6.28%) of the children had a TB contact history at admission (Table 1).

**Table 2:** Comorbidities among children under 5 years of age admitted with severe acute malnutrition to public hospitals in the city of Dire Dawa, Eastern Ethiopia, 2021 (*N* = 414)

Variables	Categories	Frequency	Percentage
Hyperthermia	No	319	77.05%
	Yes	95	22.95%
Pneumonia	No	238	57.49%
	Yes	176	42.51%
Malaria	No	407	98.31%
	Yes	7	1.69%
Vomiting	No	295	71.26%
	Yes	119	28.74%
Diarrhea	No	158	38.16%
	Yes	256	61.84%
Sepsis	No	359	86.71%
	Yes	55	13.29%
Severe anemia	No	303	73.19%
	Yes	111	26.81%
Superficial infection	No	382	92.27%
	Yes	32	7.73%
HIV/AIDS	No	394	95.17%
	Yes	20	4.83%

### 3.3 Care and treatment-related characteristics

The majority (97.83%) of the children had taken a routine medication: 94.20% of the children had taken ampicillin and 92.27% had taken gentamicin. Regarding vitamin and mineral supplementation, 43.48%, 33.09%, and 32.61% of the children had been supplied with folic acid, zinc, and vitamin A, respectively (Table 3).

**Table 3:** Care and treatment-related characteristics of children under 5 years of age admitted with severe acute malnutrition to public hospitals in the city of Dire Dawa, Eastern Ethiopia, 2021 (*N* = 414)

Variable	Categories	Frequency	Percentage
Ampicillin	No	24	5.8%
	Yes	390	94.20%
Gentamicin	No	32	7.73%
	Yes	382	92.27%
Vitamin A	No	279	67.39%
	Yes	135	32.61%
Albendazole	No	398	96.14%
	Yes	16	3.86%
Folic acid	No	234	56.52%
	Yes	180	43.48%
Measles vaccination	No	403	97.34%
	Yes	11	2.66%
Zinc	No	165	39.86%
	Yes	137	33.09%
	Not indicated	112	27.05%
ReSoMal^a^	No	210	50.72%
	Yes	204	49.28%
Paracetamol	No	251	60.63%
	Yes	163	39.37%
Blood transfusion	No	393	94.93%
	Yes	21	5.07%
Intravenous fluid	No	364	87.92%
	Yes	50	12.08%
Intravenous antibiotics	No	305	73.67%
	Yes	109	26.33%

a Rehydration solution for malnutrition.

### 3.4 Prevalence of TB among children with severe acute malnutrition

**Figure 1:**
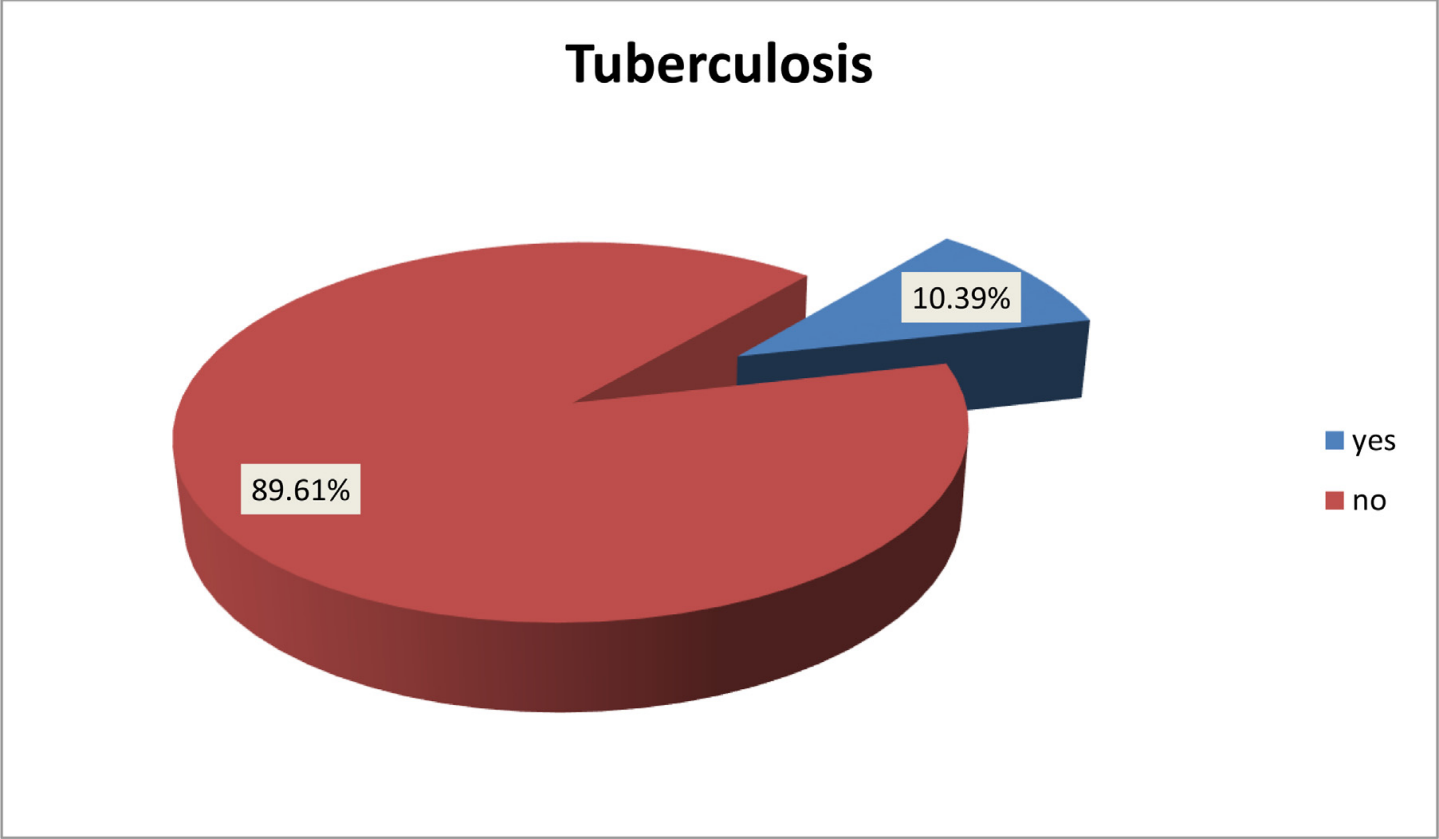
Prevalence of tuberculosis among children under 5 years of age with severe acute malnutrition admitted to public hospitals in the city of Dire Dawa, Eastern Ethiopia, 2021.

The overall prevalence of TB among under-five children admitted with SAM to the public hospitals in the city of Dire Dawa was 10.39% (95% CI 7.61–13.73%) (Figure 1). More than two-thirds (65.91%) of the children with TB had pulmonary TB, and the rest (34.09%) had extrapulmonary TB.

### 3.5 Factors associated with the prevalence of TB among children with severe acute malnutrition

In the bivariate binary logistic regression analysis, statistical significance was found for age, admission status, exclusive breastfeeding, TB contact history, hyperthermia, pneumonia, severe anemia, HIV/AIDS, diarrhea, level of consciousness, taking intravenous fluid, and immunization status. Of these, admission status, HIV/AIDS, pneumonia, TB contact history, and immunization status remained statistically significant in the multivariate logistic regression analysis at *P* < 0.05.

According to the multivariate binary logistic regression analysis, the odds of having TB were 2.5 times higher among under-five children with SAM with a repeat admission as compared to newly admitted children with SAM (AOR 2.5, 95% CI 1.08–6.07). The odds of having TB were 3.6 times higher among under-five children who had a TB contact history as compared to those without this history (AOR 3.58, 95% CI 1.21–10.6). The odds of being infected with TB were 2.8 times higher among children who had pneumonia as a comorbidity than among those without pneumonia (AOR 2.8, 95% CI 1.29–6.23). Being HIV-positive increased the odds of having TB by 4.4 times (AOR 4.41, 95% CI 1.29–15.13). The odds of having TB were reduced by 81% for those children who had been immunized as compared to the unimmunized (AOR 0.19, 95% CI 0.08–0.43) (Table 4).

**Table 4:** Factors associated with TB prevalence among children under 5 years of age admitted with severe acute malnutrition to public hospitals in the city of Dire Dawa, Eastern Ethiopia, 2021 (*N* = 414)

Variable	Categories	Tuberculosis		COR (95% CI)	AOR (95% CI)
		No	Yes		
Age	<24 months	241	21	1	1
	≥24 months	130	22	1.94 (1.02, 3.66)	1.5 (0.69, 3.28)
Admission status	New	311	27	1	1
	Repeat	60	16	3.07 (1.56, 6.04)	2.5 (1.08, 6.07)*
Exclusive breastfeeding	No	121	27	1	1
	Yes	250	16	0.28 (0.14, 0.55)	0.47 (0.22, 1.04)
TB contact history	No	354	34	1	1
	Yes	17	9	5.51 (2.28, 13.30)	3.58 (1.21, 10.6)*
Hyperthermia	No	289	30	1	1
	Yes	82	13	1.5 (0.76, 3.06)	1.7 (0.72, 4.03)
Pneumonia	No	223	15	1	1
	Yes	148	28	2.81 (1.45, 5.44)	2.8 (1.29, 6.23)**
Severe anemia	No	281	22	1	1
	Yes	90	21	2.98 (1.56, 5.67)	2.19 (0.99, 4.83)
HIV/AIDS	No	358	36	1	1
	Yes	13	7	5.34 (2.00, 14.27)	4.41 (1.29, 15.1)**
Diarrhea	No	148	10	1	1
	Yes	223	33	2.19 (1.04, 4.58)	2.02 (0.84, 4.84)
Level of consciousness	Conscious	310	28	1	1
	Unconscious	61	15	2.72 (1.37, 5.39)	1.76 (0.74, 4.18)
IV fluids	No	332	32	1	1
	Yes	39	11	2.92 (1.36, 6.26)	1.49 (0.56, 3.93)
Immunization status	Immunized	89	29	0.15 (0.77, 0.301)	0.19 (0.08, 0.43)***
	Not immunized	282	14	1	1

AOR, adjusted odds ratio; CI, confidence interval; COR, crude odds ratio; IV, intravenous; TB, tuberculosis. *Significant, *P* < 0.05; **significant, *P* < 0.01; ***significant, *P* < 0.001.

## 4 Discussion

The overall prevalence of TB among under-five children admitted with SAM to public hospitals in the city of Dire Dawa, Eastern Ethiopia was 10.39% (95% CI 7.61–13.73%), which is in line with the results of a study conducted in Karnataka, India (10.34%) (S et al., 2019). However, the prevalence found in this study is higher than those reported in studies conducted in Lusaka, Zambia (1.58%) (Munthali et al., 2017) and Nepal (4.67%) (Thakur et al., 2020). This might be because the study conducted in Nepal included only bacteriologically confirmed TB cases, which may underestimate the prevalence of TB. In the current study, TB was diagnosed by chest X-ray and clinically by physicians. Another factor contributing to the increased TB prevalence in the current study might be the lifestyle habits of the people: in Ethiopia people share everything, which might increase the transmission of TB. In contrast, the prevalence in the present study was lower than that reported in a study conducted in Pakistan (36.9%) (Khalil et al., 2020). This discrepancy could be due to Pakistan being among the eight highest TB prevalent countries, which contribute two-thirds of the global TB prevalence according to the WHO 2020 report (Shiferaw et al., 2018).

The current study showed that repeated admission increased the odds of having TB among under-five children with SAM. Children under 5 years of age repeatedly admitted with SAM would have compromised immunity, which increases the development of active TB.

In line with studies conducted in Nigeria (Attah et al., 2018), and India (S et al., 2019), the current study showed that the odds of having TB increased with a TB contact history. This is because contact with TB patients is the main transmission mechanism of TB. Having contact with TB cases generally leads to a 50% chance of developing TB (Kliegman et al., 2020) . Similar to a previous study conducted in Bangladesh (Chisti et al., 2014), pneumonia was found to increase the risk of developing TB in the current study. Pneumonia increases the reactivations of latent TB and it also provides a fertile environment to develop active TB (Fatahi-Bafghi, 2021; Oliwa et al., 2015).

The odds of having TB were reduced by immunization, which is supported by a study done in Pakistan (Khalil et al., 2020). The Bacillus Calmette–Guérin (BCG) vaccine is the proven way to prevent TB, thus, TB prevalence is expected to be reduced among immunized children (Darrah et al., 2020). The odds of having TB were higher in children with HIV/AIDS than in those without HIV/AIDS, which is supported by studies conducted in Zambia (Munthali et al., 2017). This might be because having HIV/AIDS may worsen pre-existing under-nutrition, thereby leading to a marked reduction in the immune system, which again might increase susceptibility to TB. HIV infection increases the risk of developing active TB by 10 times (Mhango et al., 2021).

This study has some important limitations. Since the study was based on secondary data, some important variables were not included in this analysis, which might have affect the result. Additionally, children diagnosed clinically by physician judgement were categorized as TB cases, which might have led to an overestimation of the result.

In conclusion, the prevalence of TB among under-five children with SAM was found to be high in the public hospitals of Dire Dawa City, Eastern Ethiopia. Admission status, HIV/AIDS, pneumonia, TB contact history, and immunization status were variables significantly associated with the prevalence of TB among children admitted with SAM to the public hospitals in the city of Dire Dawa, Eastern Ethiopia. Integrative approaches that consist of TB screening and prevention in existing nutritional rehabilitation centers should be implemented.

## Author contributions

RNH, KAA, and MAT conceived and designed the study, performed the analysis, and drafted the manuscript. All authors critically reviewed the manuscript for important intellectual content and contributed to the final approval of the version to be submitted.
